# Determination of the Sterilization Dose of Gamma-Ray Irradiation for Polyvinyl Alcohol-Collagen-Chitosan Composite Membrane as a Material for Periodontal Regenerative Surgery

**DOI:** 10.1055/s-0043-1761186

**Published:** 2023-06-27

**Authors:** Agus Susanto, Ira Komara, Maria Theresia Beatrix, Fajar Lukitowati, Amaliya Amaliya, Ina Hendiani, Aldilla Miranda

**Affiliations:** 1 Department of Periodontics, Faculty of Dentistry, Universitas Padjadjaran, Bandung, Indonesia; 2 Research Center for Radiation Process Technology—National Research and Innovation Agency (NRIA), Indonesia

**Keywords:** PVA–collagen–chitosan, sterilization, gamma-ray irradiation

## Abstract

**Objective** Membrane sterility is very necessary considering its function as an implant material. Therefore, this research aims to determine the dose of gamma-ray irradiation for the sterilization of polyvinyl alcohol (PVA)–collagen–chitosan composite membranes used as regenerative surgery materials.

**Materials and Methods** A total of 100 pieces of the composite membranes were prepared in a size of 2.0 × 1.5 cm by mixing 7.5% PVA, 3% collagen, and 2% chitosan using the film casting method in three batches. Furthermore, the bioburden test was performed to determine the initial microbial count in the sample by following ISO 11737-1. The results were used to ascertain the dose of gamma-ray irradiation on the sample according to ISO 11137-2. The dose verification test was then performed at the sterility assurance level 10 ^−6^ .

**Results** The average result of the bioburden test from three batches was 6.6 colony forming unit; hence, the verification dose was 4.8 kGy. In the verification dose test, since there was only one contaminated sample, the sterility dose test was continued.

**Conclusion** The sterile gamma-ray irradiation dose for PVA–collagen–chitosan composite membrane was 17.1 kGy.

## Introduction

The goal of regenerative periodontal therapy is to completely restore the teeth-supporting tissues that have been lost due to periodontal tissue inflammation or destruction. Various periodontal regenerative surgical treatments are continuously developed in various ways such as the use of materials capable of assisting tissue regeneration, for example, barrier membranes, bone grafts, osteoconductive materials, and growth factors. One surgical treatment approach that is often applied and has proven successful in increasing tissue regeneration is the *guided tissue regeneration* (GTR) and *guided bone regeneration* (GBR) methods. 1 2 3 4 Membrane barrier usage was commenced when the GTR concept was introduced, with its main function to inhibit epithelial cells migration by placing the membrane between the flap and root surface, thereby preventing connective tissue contact and isolating the cells to restore the periodontal ligament, cementum, and connective tissue. 5 6

In general, barrier membranes are divided into two, namely nonresorbable and resorbable. The use of resorbable membranes in recent years has increased as this reduces the need for a second surgical procedure. Additionally, their advantages include reduced patient discomfort, bioactive properties, and ease of application. The disadvantages are unpredictable resorption patterns associated with hydrolytic or enzymatic degradation processes and possible inflammation associated with the degradation. 7 Some of the widely used resorbable membrane materials are polylactic acid, polyglycolic acid, poly lactic-coglycolic acid copolymer, and collagen membrane. 8 9 10

The ideal requirements of membranes for GTR must meet several principles, namely being acceptable to body tissues, not causing tissue toxicity, not activating the immune response or causing acute inflammation (biocompatibility), acting as a barrier and removing certain types of cells (cell exclusion), preventing the growth of epithelial cells and encapsulation of material (tissue integration), can create and maintain space adjacent to the root surface so as to allow the growth of tissue from the periodontal ligament (space-making), and is easy to apply. 11 12 One of the requirements for periodontal surgical materials is their sterility; therefore, the membrane to be used for GTR or other operations should be sterilized first. 13 After the 1980s, nonconventional surgical methods were developed, and with advances in the medical field new alternative sterilization methods have been created such as gamma radiation sterilization. 14

The main source of radiation is gamma rays produced from the radioactive material Cobalt 60, which can be used to illuminate thick objects due to its high penetrating power. Gamma-ray irradiation is often referred to as cold sterilization. 15 Radiation processes can be used to modify polymer properties and produce two results, namely the occurrence of cross-linking or chain breaking, but chain breaking is an undesirable result. The advantage of sterilization with gamma ray irradiation include not leaving residue on objects, not changing the tissue structure due to being a cold sterilizer, effectively killing microorganisms to a certain extent, and possession of high penetrating power. 12 16 17 However, gamma-ray irradiation can affect materials' physicochemical and biological properties by causing cross-linking or breaking of bonds. 18 The dose of gamma irradiation required to sterilize medical equipment according to ISO 11137 is 15 or 25 kGy. 19 20 21 22 Based on the ISO 11137 guidelines, gamma-ray irradiation sterilization dose is ascertained through three stages, namely initial microbial test (bioburden), determination of verification dose, and sterility test. 22

Determination of the right gamma irradiation dose tends to produce an ideal membrane under sterile conditions during production, indicating that it can be used directly without changing the quality. Therefore, this research aims to determine the gamma-ray irradiation dose for the sterilization of PVA–collagen–chitosan composite membranes.

## Materials and Methods

### Materials and Equipment

The materials used were collagen extracted from bovine tendons, medical-grade chitosan obtained from shrimp shells, polyvinyl alcohol (Merck), acetic acid (Merck), sodium hydroxide (NaOH) (Merck), tryptic soy agar (TSA; Merck), fluid thioglycolate medium (FTM; BD), and sterile distilled water. Meanwhile, the equipment employed were gamma rays (Gammacell 220), analytical balance (Acculab), glass (measuring cup, Erlenmeyer flask, test tube, and petri dish), magnetic stirrer (Fischer Scientific), autoclave (Hiramaya), laminar airflow (Envar), bunsen burner, and polyethylene plastic.

### Polyvinyl Alcohol -Collagen-Chitosan Membrane Composite Manufacture

A 3% collagen solution from overnight acetate homogenization, 2% chitosan solution, and PVA solution with a concentration of 7.5% was made. The procedure for preparing the membrane was according to Susanto et al (2021). The three solutions of PVA–collagen–chitosan in a ratio of 1:1:1 were mixed, followed by an overnight homogenization process. The composite was then poured into an acrylic mold measuring 7.5 × 7.5 cm up to 10 g and dried at room temperature for 3 to 4 days. Afterward, the dry membrane was immersed in NaOH solution for 1 hour, washed until alkaline-free (neutral pH), and frozen in a deep freezer before drying under pressure (lyophilization) for 24 hours. The membrane was dried by lyophilization or freeze drying with a freeze dryer for 4 hours.

### Determination of Initial Microbial Count (Bioburden Test)

The bioburden test determines the initial number of microbes in the sample following the procedure in ISO 11737-1: Sterilization of health care products—Microbiological Methods—Part 1: Determination of a population of microorganism on products. 23 Up to 30 samples of PVA–collagen–chitosan membrane in a 2 × 1.5 cm size from three different production batches were selected (from every production batch 10 samples) and each sample was put into 5 mL of sterile distilled water. Furthermore, the microorganisms were transferred by vortex extraction for ± 5 minutes and allowed to stand for 1 hour. All distilled water was poured into three sterile Petri dishes, then TSA was added to each petri dish and gently shaken. This was allowed to solidify and later incubated at 37°C for 4 to 5 days. The number of colonies growing on the TSA media after incubation was observed and recorded, followed by counting and recording the number of microbes growing on each TSA medium after incubation was counted and recorded. Furthermore, microbes from the three batches were summed and averaged.

### Determination of Sterilization Dose according to ISO 11137

The estimated radiation dose produced a sterility assurance level (SAL) of 10 ^−2^ , and then, the sterilization dose was determined based on the table on ISO 11137-2 ( Table 1 ) and based on the initial number of microorganism populations from the bioburden test, and we determined doses based on SAL 10 ^−6^ . 24

**Table 1:** Radiation dose (kGy) required to achieve a given SAL for an average bioburden greater than or equal to 1,0, which has the standard distribution of resistances (SDR) 24

Average bioburden (CFU)	Sterility assurance level				
	10 ^−2^	10 ^−3^	10 ^−4^	10 ^−5^	10 ^−6^
01.0	3.0	5.2	8.0	11.0	14.2
1.5	3.3	5.7	8.5	11.5	14.8
2.0	3.6	6.0	8.8	11.9	15.2
…....	…....	…....	…....	…....	…....
…....	…....	…....	…....	…....	…....
4.0	4.3	6.8	9.7	12.9	16.2
4.5	4.4	7.0	9.9	13.1	16.4
5.0	4.5	7.1	10.0	13.2	16.6
5.5	4.6	7.2	10.2	13.4	16.7
6.0	4.7	7.3	10.3	13.5	16.9
6.5	4.8	7.4	10.4	13.6	17.0
**7.0**	**4.8**	7.5	10.5	13.7	**17.1**
7.5	4.9	7.6	10.6	13.8	17.2
8.0	5.0	7.7	10.7	13.9	17.3
8.5	5.1	7.8	10.8	14.0	17.4
9.0	5.1	7.8	10.8	14.1	17.5
9.5	5.2	7.9	10.9	14.1	17.6
10	5.2	8.0	11.0	14.2	17.6
11	5.3	8.1	11.1	14.3	17.8
…....	…....	…....	…....	…....	…....
…....	…....	…....	…....	…....	…....
900000	21.0	24.7	28.5	32.3	36.2
950000	21.1	24.8	28.5	32.4	36.3
1000000	21.2	24.9	28.6	32.5	36..3

Abbreviation: CFU, colony forming unit.

Source: Table copied from Table 5. ISO 11137–2 by retrieving only part of the data.

### Gamma-Ray Irradiation

A total 100 samples of PVA–collagen–chitosan membranes from three production batches measuring 2 × 1.5 cm were put into polypropylene plastic bags and then irradiated using gamma rays with the obtained sterilization dose. In total, 100 samples from three different production batches had been marked with a sterilization indicator (indicators for gamma/E-beam sterilization), and dose measurement with the Harwell Amber 3042 dosimeter used the same dose as above.

### Test Sterilization Dose according to ISO 11737-2

A total of 100 samples measuring 2 × 1.5 cm that had been irradiated with a sterilization dose were selected using sterile tweezers and dropped into a test tube containing FTM. Furthermore, they were set in a standing position and submerged in the medium. The media was then incubated at 37°C for 14 days. The presence of samples that changed from clear to cloudy was noted. After receiving the sterilization dose, where the results of the above-mentioned sterility test produced a positive value < 2, the dose was determined using SAL = 10 ^−6^ . The SAL guarantee level is the probability of a microorganism surviving in the production unit poststerilization. 25

### Data Analysis

The data obtained were quantitative and qualitative, where the quantitative was in the form of the number of microbes before gamma-ray irradiation. Qualitative data in the form of sterility after gamma irradiation were analyzed based on Table 2 of the bioburden test results to determine the sterilization dose of the PVA–collagen–chitosan composite membrane.

**Table 2:** Average initial microbial test results

Repeat	Total microbes (CFU)		
	Batch 1	Batch 2	Batch 3
1	6	7	4
2	8	3	9
3	10	5	4
4	4	6	6
5	2	5	4
6	11	7	1
7	6	6	5
8	7	5	30
9	12	6	3
10	4	5	6
Average	7	5.5	7.2
Overall average	(7 + 5.5 + 7.2)/3 = **6.6**		

Abbreviation: CFU, colony forming unit.

## Results and Discussion

Based on ISO 11137 guidelines, the sterilization dose can be determined in three stages, namely initial microbial test (bioburden), determination of verification dose, and sterility test. The results of the bioburden test on the PVA–collagen–chitosan membrane are shown in Table 2 . The average amount of initial microbial contamination on the membrane was 7, 5.5, and 7.2 CFU (colony forming unit), and the overall average was 6.6 CFU as presented in the table.

According to the ISO 11137-2 guidelines, if the average bioburden value is not listed in the table, the bioburden level value above is used to calculate the verification dose. There was no bioburden test result of 6.6 CFU in Table 1 , and then the value used at the level above to determine the verification dose was 7.0 CFU. Based on Table 1 , the average bioburden value was 7.0 and the verification dose was 4.8 kGy.

Furthermore, a sterility test was performed using 100 samples of PVA–collagen–chitosan membranes irradiated with 4.8 kGy gamma rays. Before radiation, the sample was measured with a Harwell Amber 3042 dosimeter to determine the real or absorbed dose received by the sample. The dosimeter measurement results showed that the absorbed dose was 4.78 kGy.

**Fig. 1:**
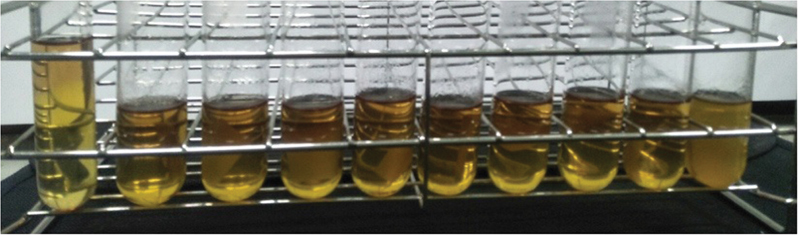
( **A** ) Nine sample tubes where one (far right) is cloudy due to microbial growth. ( **B** ) The leftmost tube is the control group tube.

The results of the sterility test on samples irradiated with a verification dose can be seen in Fig. 1 . Furthermore, out of 100 samples, only one was contaminated or not sterile. Based on the ISO 11137 guidelines, a verification dose is acceptable if out of 100 samples irradiated at this verification dose, there are only a maximum of 2 unsterile samples. In this experiment, there was one unsterile sample; hence, the verification dose was received and then used to determine the sterilization dose. Based on the results, it was concluded that the sterilization dose of gamma-ray irradiation for PVA–collagen–chitosan membrane at the SAL 10 ^−6^ was 17.1 kGy.

The results showed that gamma-ray irradiation of PVA–collagen– chitosan membranes with a verification dose of 4.8 kGy was effective in sterilizing the membranes. This is indicated by only one sample experiencing microbial growth (turbidity occurs) out of the 100 tested for sterility.

Based on ISO 11137 guidelines, the determination of the gamma-ray irradiation dose was performed in three stages, namely initial microbial test (bioburden), determination of verification dose, and sterility test. An initial microbial test was performed to determine the amount of microbial contamination present on the membrane. The lesser the microbial contamination, the smaller the dose of irradiation required to sterilize the membrane. Based on the results of the PVA–collagen– chitosan membrane bioburden test from three batches shown in Table 2 , the average values were 7, 5.5, and 7.2 CFU; hence, the overall average yield was 6.6 CFU. The result was considered as the amount of contamination present on the membrane and also applied to determine the verification dose using Table 1 as a reference.

According to ISO 11137, if the average bioburden value is not in Table 1 , the bioburden level value above is used to calculate the verification dose. The bioburden value used to determine the verification dose was 7.0 because 6.6 was not presented. At 7.0 bioburden and SAL 10 ^−2^ , the verification dose was 4.8 kGy. Determination of the verification dose by microbial testing according to the existing contaminants is important since excessive irradiation can damage the membrane. The most vital aspect of sterilization using gamma-ray irradiation is the tolerance of a product (in this case the PVA–collagen–Chitosan membrane) to radiation. 16 The specified dose is subjected to a sterilization test as evidence that the dose can effectively sterilize the membrane.

The advantages of sterilization with gamma-ray irradiation include not leaving residue on the material, not changing the tissue structure due to being cold sterilization, effectively killing microorganisms to a certain extent, and possession of high penetrating power. 12 16 Gamma irradiation functions as physical decontamination as energy from the rays destroy pathogens that can contaminate the membrane. The energy induced by photons is capable of altering biological molecules and cellular structures. Radiation processes can be used to modify polymer properties and produce two results, namely the occurrence of cross-linking or chain breaking, but chain breaking is an undesirable result. Gamma-ray irradiation can break hydrogen bonds between two macromolecules. The dose used was too high and could cause the degradation of the chitosan chain. This affected the physicochemical and biological properties of the membrane. 18

A total of 100 membrane samples were packed into polypropylene plastic bags and prepared to be irradiated at a dose of 4.8 kGy with gamma rays after determining the verification dose. Previously, they were measured with Harwell Amber 3042 dosimeter to determine the actual or absorbed dose that the samples received. According to the ISO 11137 guidelines, the verification dose is acceptable if the absorbed dose does not exceed 10% (4.8 kGy) and is not lower than 90% of the target dose. Harwell Amber 3042 dosimeter measurement results showed that the real dose received by the samples was 4.78 kGy, meaning the absorbed dose was still within the guidance range according to ISO 11137.

Furthermore, after irradiation, a sterility test was performed by immersing the samples in a solution of FTM for 14 days, followed by incubation at a temperature of 37°C. The presence of turbidity indicates that there is microbial contamination. The results of the sterility test on samples irradiated with a verification dose are presented in Fig. 1 . It can be seen that from 100 samples only one was not sterile (microbes grow). According to the ISO 11137 guidelines, a verification dose is accepted if the unsterile samples out of the total 100 irradiated at this verification dose do not exceed 2 (due to microbial growth). In this experiment, only one sample ( Fig. 1 ) was not sterile; hence, the verification dose was acceptable and used to determine the sterilization dose.

The sterility dose was determined using the SAL 10 ^−6^ after receiving the verification dose. SAL 10 ^−6^ means that out of 1 million sterile membrane production, only a maximum of 2 nonsterile products are allowed. SAL 10 ^−6^ was selected due to being one of the requirements for the material to be in direct contact with body fluids and internal organs. 26 Based on ISO 11137 guidelines, the sterilization dose with SAL 10 ^−6^ is 17.1 kGy ( Table 1 ), which is the dose used to sterilize the PVA–collagen–chitosan membrane to be produced in large quantities. 21

## Conclusion

Based on the results of the determination of the bioburden of the PVA–collagen–chitosan composite membranes, the initial contamination was 7.0 CFU and the verification dose obtained was 4.8 kGy. Meanwhile, the gamma-ray dose for the sterilization of the membrane at SAL 10 ^−6^ was 17.1 kGy. Recommendations for future research include in vitro studies of the physical and mechanical strength tests and in vivo studies of biocompatibility, tissue integration, and biodegradation of the PVA–collagen–chitosan membrane after gamma radiation sterilization.
